# Supplementary material for: Intact RNA structurome reveals mRNA structure-mediated regulation of miRNA cleavage in vivo
Source: Nucleic Acids Res. 2020 Jul 11;48(15):8767–81. doi: 10.1093/nar/gkaa577 (PMC7470952; doi:10.1093/nar/gkaa577)
Supplement: gkaa577_Supplemental_File [file gkaa577_supplemental_file.pdf]

Intact RNA structurome reveals mRNA structure-mediated regulation of miRNA cleavage *in vivo*

Minglei Yang[1,*], Hugh C. Woolfenden[1,*], Yueying Zhang[1,*], Xiaofeng Fang[1,*], Qi Liu[1], Maria Louisa Vigh[2], Jitender Cheema[1], Xiaofei Yang[1], Matthew Norris[1], Sha Yu[1,3], Alberto Carbonell[4], Peter Brodersen[2], Jiawei Wang[3], Yiliang Ding[1,†]

Supplementary Information

Figure S1. The illustration of the spurious chemical reactivity signals caused by degradation products.

Figure S2. Establishment of CAP-STRUCTURE-seq.

Figure S3. Overview of cleavage efficiency calculation.

Figure S4. Overview of CAP-STRUCTURE-seq libraries.

Figure S5. Library reproducibility and correlation.

Figure S6. CAP-STRUCTURE-seq provides the complete map of the 18S rRNA *in vivo* structure at nucleotide resolution.

Figure S7. Meta-properties of structure features confirm the validity of CAP-STRUCTURE-seq.

Figure S8. Correlations for various flank lengths around the target site.

Figure S9. Validation of TAM functionality by a designed structure assay.

Table S1. The list of oligos and primers sequences.

Table S2. CAP-STRUCTURE-seq constraints improve the structure prediction of structure in the 18S rRNA.

Table S3. The list of CE values for the previously reported target genes.

Supplementary Methods. The detailed calculation of miRNA cleavage efficiency.

References

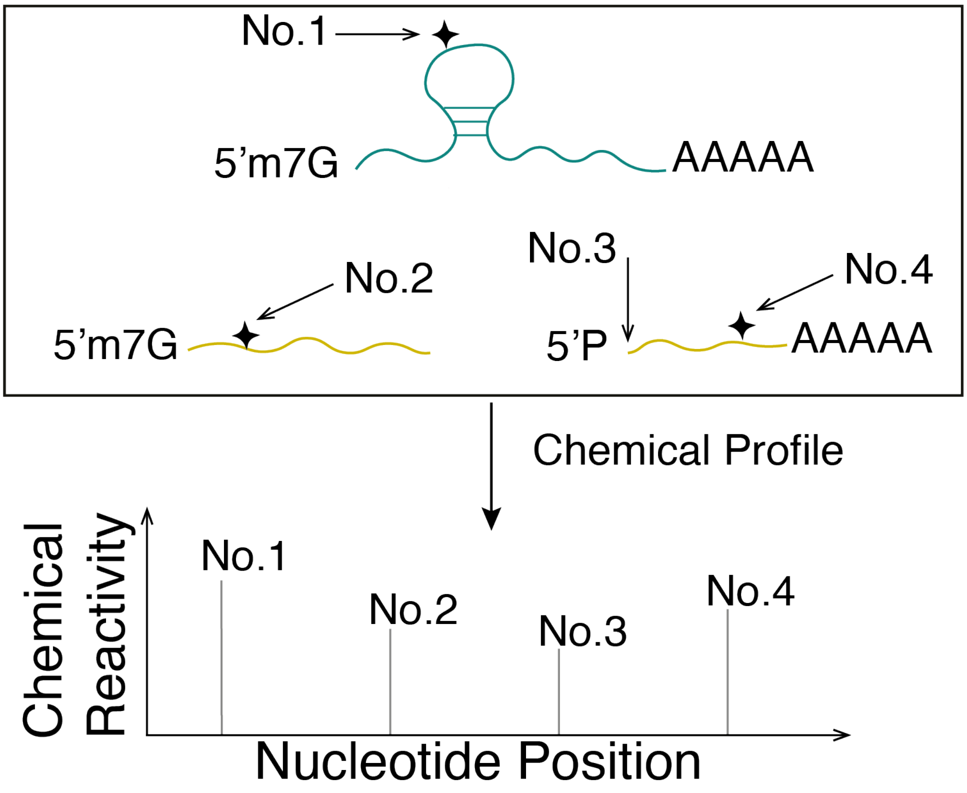

Figure S1. The illustration of the spurious chemical reactivity signals caused by degradation products. The final chemical reactivity contains the RNA structure information from both degraded RNAs and intact RNAs. Additionally, degraded mRNAs are capable of introducing false positive signals in the reverse transcription stalling methods. For example, the No.3 SHAPE reactivity is not caused by chemical modification but from a degradation event.

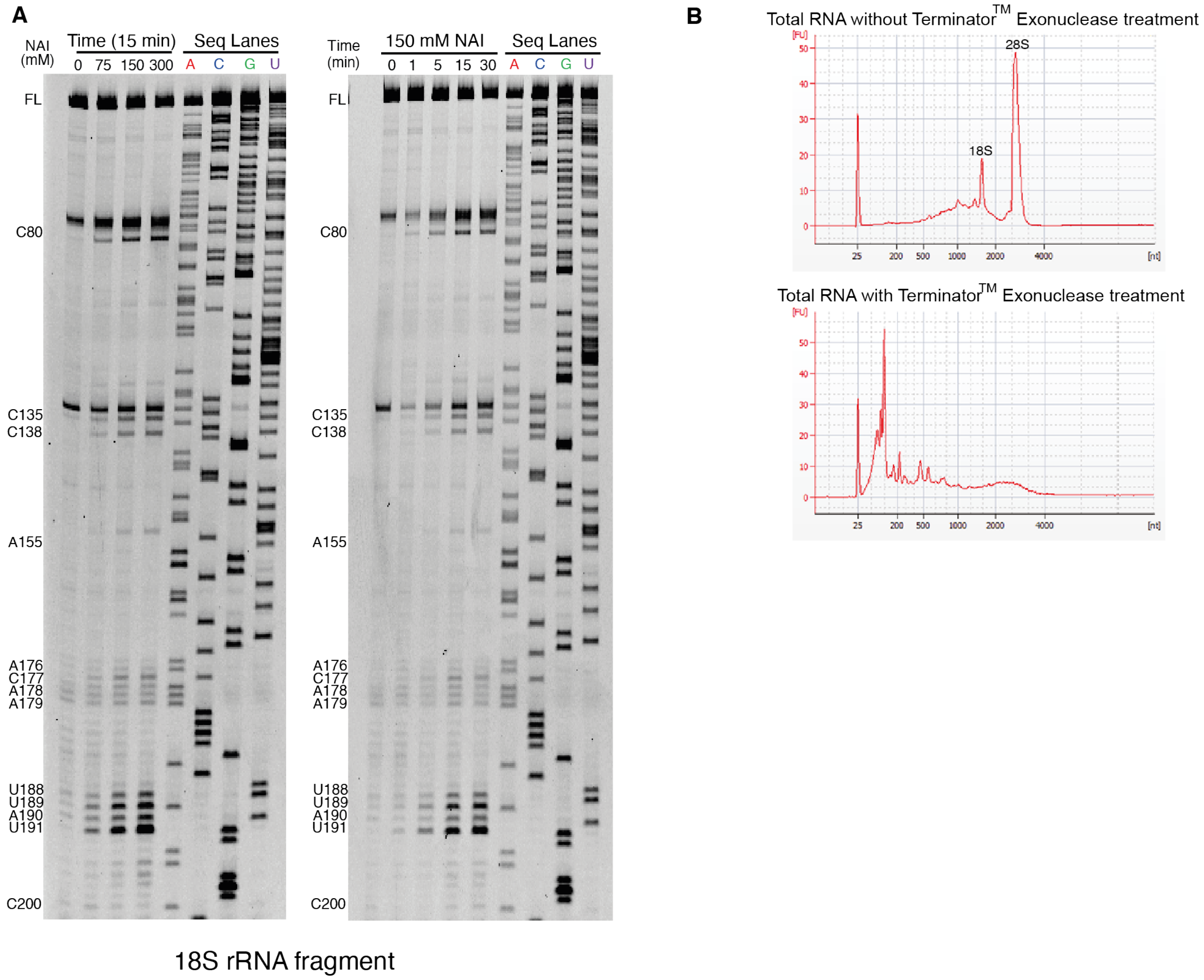

Figure S2. Establishment of CAP-STRUCTURE-seq. A, NAI was titrated to achieve single hit kinetics in structure probing. Gel analysis of 18S rRNA structure probing in the presence of 1 µg of total *A. thaliana* RNA at NAI concentration of 75 mM, 150 mM and 300 mM (left). Time course analysis of *in vivo* SHAPE modification of 18S rRNA in *A. thaliana* etiolated seedlings with durations of 1 min, 5 min, 15 min and 30 min (right). FL, full length. Seq Lanes, sequencing lanes**. B,** Bioanalyzer assay indicated Terminator enzyme could digest 5’ phosphate transcripts. The peaks of 18S and 28S rRNAs, which have 5’ phosphate, decreased dramatically after Terminator treatment (bottom) compared to non-treatment (top).

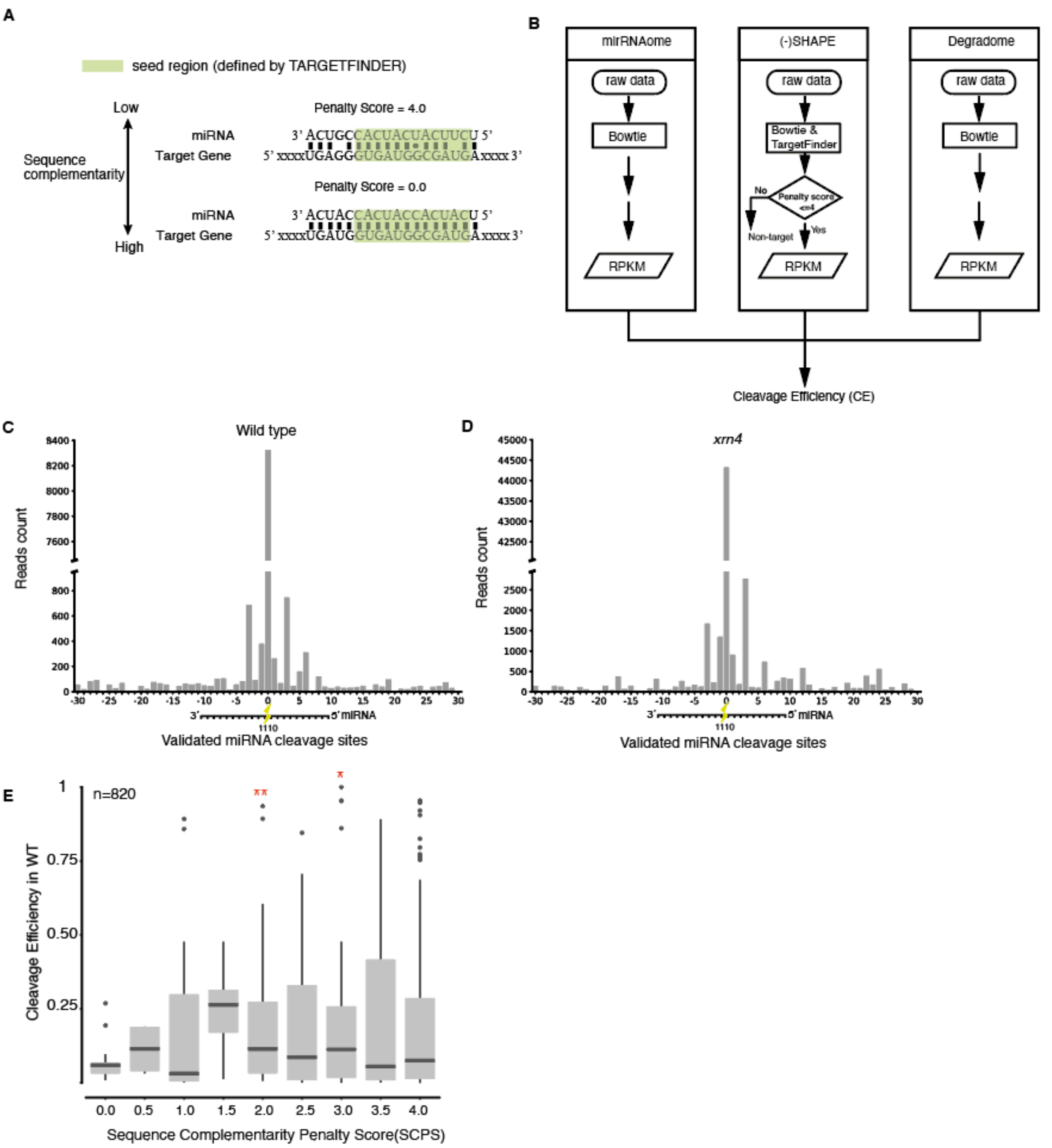

Figure S3. Overview of cleavage efficiency calculation. A, Sequence complementarity penalty score (SCPS), generated by TargetFinder (1), is inversely related to sequence complementarity. A SCPS score of zero is equivalent to perfect complementarity between the miRNA and the target site. We predicted the plant miRNAs targets of those expressed miRNAs by *TargetFinder*. Then, we chose the targets which overlapped with all the validated miRNA targets from four different genome-wide studies (23–26). **B,** The pipeline for combining miRNAome, (-)SHAPE and Degradome to calculate transcriptome-wide Cleavage Efficiency (CE) *in vivo*. Detailed explanation is in the Methods and the Supplementary Methods. **C** and **D,** Degradome reads distribution in WT (**C**) and *xrn4* mutant (**D**) around identified miRNA cleavage sites (2). The zero position on the horizontal axis indicates the miRNA cleavage sites at the tenth position of miRNA complementary sites (as illustrated beneath the horizontal-axis). **E,** Target site sequence complementarity does not linearly correlate with miRNA cleavage efficiency (CE). Some target sites with mismatches (SCPS=2.0 and 3.0) cleave more efficiently

than perfectly matched target sites (SCPS=0). Mann-Whitney-U significance tests were performed. 820 positive CE target sites were analyzed.

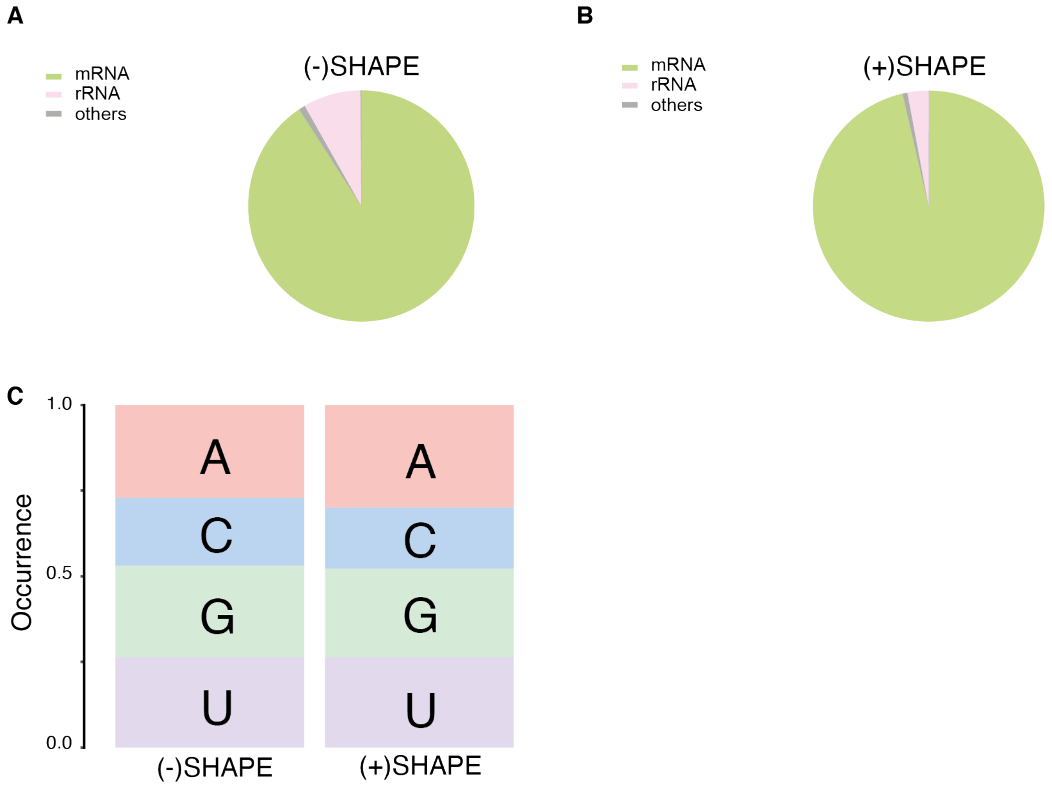

Figure S4. Overview of CAP-STRUCTURE-seq libraries. A, RNA types for the (-)SHAPE library. The pie chart depicts the frequency of different classes of RNA species present in the (-)SHAPE datasets using RPKM values. **B,** RNA types for the (+)SHAPE library. The pie chart depicts the frequency of different classes of RNA species present in the (+)SHAPE datasets using RPKM values. **C,** SHAPE modification shows no nucleotide preference between the (-)SHAPE and the (+)SHAPE libraries. The nucleotide corresponds to the modified nucleotide, which is one position towards the 5' end of the transcript from the reverse transcriptase stalling position.

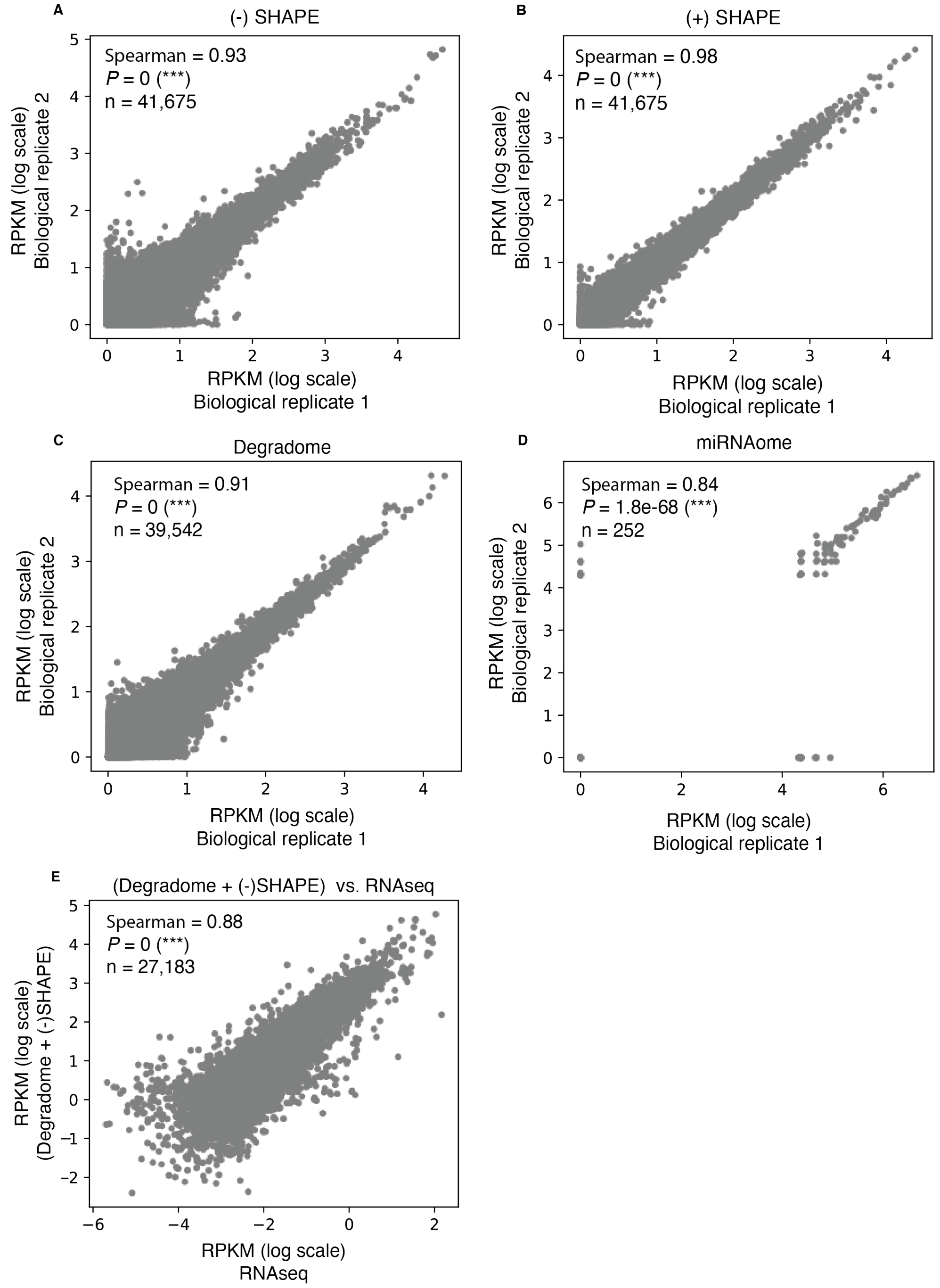

Figure S5. Library reproducibility and correlation. A, Correlation between the RPKM of (-)SHAPE libraries across the transcriptome in biological replicate 1 and 2. **B,** Correlation between the RPKM of (+)SHAPE libraries across the transcriptome in biological replicate 1 and 2. **C,** Correlation between the RPKM of Degradome libraries across the transcriptome in biological replicate 1 and 2. **D,** Correlation between the RPKM of miRNA libraries in biological replicate 1 and 2**. E,** Correlation between the RPKM of (-)SHAPE plus Degradome libraries and canonical RNA-seq libraries across the transcriptome.

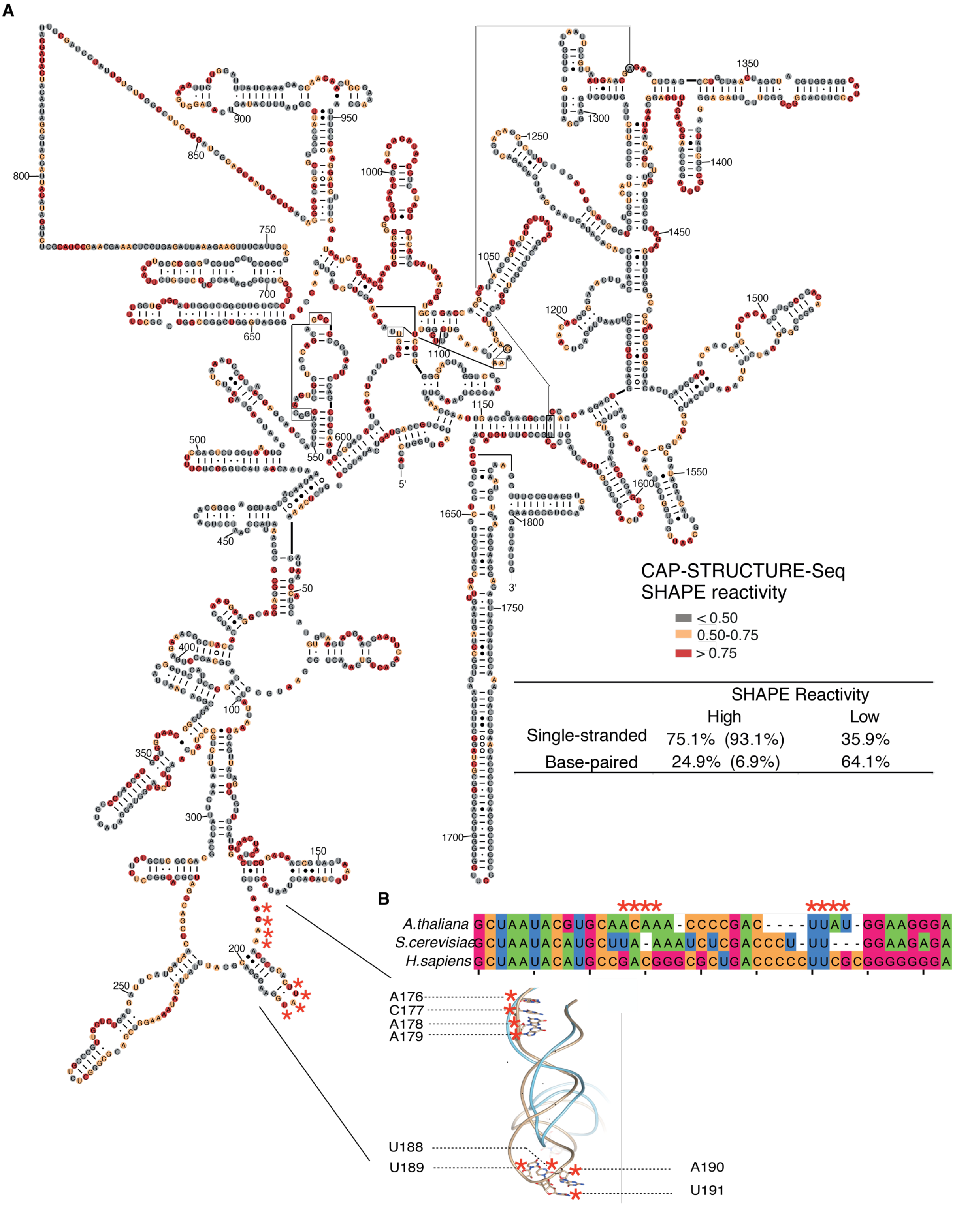

Figure S6. CAP-STRUCTURE-seq provides the complete map of the 18S rRNA *in vivo* structure at nucleotide resolution. A, The complete 18S rRNA (length 1,808 nt) phylogenetic structure is colour-coded according to the SHAPE reactivity generated from CAP-STRUCTURE-seq (SHAPE reactivity >0.75 marked in red; SHAPE reactivity 0.5–0.75 marked in orange; SHAPE reactivity < 0.5 marked in grey). The table quantifies the correspondence between the 18S rRNA phylogenetic structure and the high and low reactivity groups. In the entire 18S rRNA (length = 1,808 nt), 75.1% of nucleotides that show high *in*

vivo SHAPE reactivity in our data set correspond to single-stranded regions in the phylogenetic structure (true positive), whereas, 64.1% of the nucleotides that show low *in vivo* SHAPE reactivity correspond to base-paired regions in the phylogenetic structure (true negative). The 35.9% of the nucleotides that show low *in vivo* SHAPE reactivity but correspond to single-stranded regions in the phylogenetic structure (false negative) are presumably protected by either ribosomal proteins or non-base-pairing tertiary RNA structure. Of the 24.9% reactive nucleotides that are annotated as base-paired in the phylogenetic structure (false positive), 75% of these nucleotides are positioned either at the end of a helix or adjacent to a helical defect such as a bulge or loop. These locations are known to lead to structural flexibility(3) **B,** Sequence alignment of *A. thaliana*, *S. cerevisiae* and *H. sapiens*, shows that the non-conserved nucleotides (red asterisks) have high SHAPE reactivities in *A. thaliana*. These nucleotides are single-stranded in the corresponding region of human (brown, PDB: 4V6X) and yeast (cyan, PDB: 4v7r) 18S rRNA. The crystal structures are presented by Chimera with the nucleotides labelled according to the *A. thaliana* location.

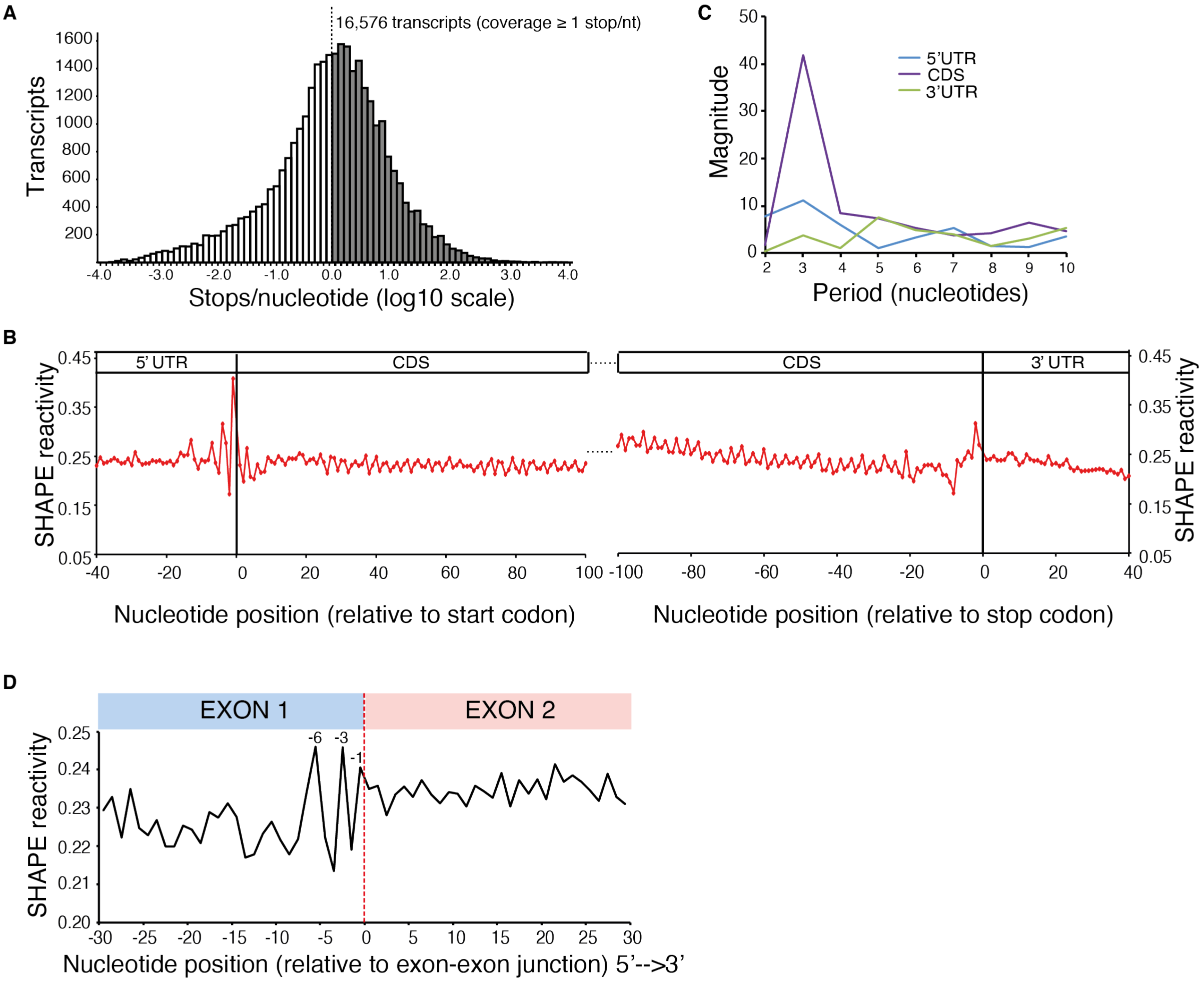

Figure S7. Meta-properties of structure features confirm the validity of CAP-STRUCTURE-seq. A, Over 16,000 transcripts in the (+)SHAPE library have an average reverse transcriptase (RT) stops per nucleotide of at least one. The number of transcripts as a function of the average RT stops divided by the length of that transcript is shown. **B,** SHAPE reactivity across the 5' UTR, the CDS and the 3' UTR. mRNAs were aligned by their start/stop codons (vertical black lines). **C,** The triplet-periodicity is present in the CDS region but absent from both 5' UTR and 3' UTR. **D,** Average SHAPE reactivities across transcript exon–exon junctions.

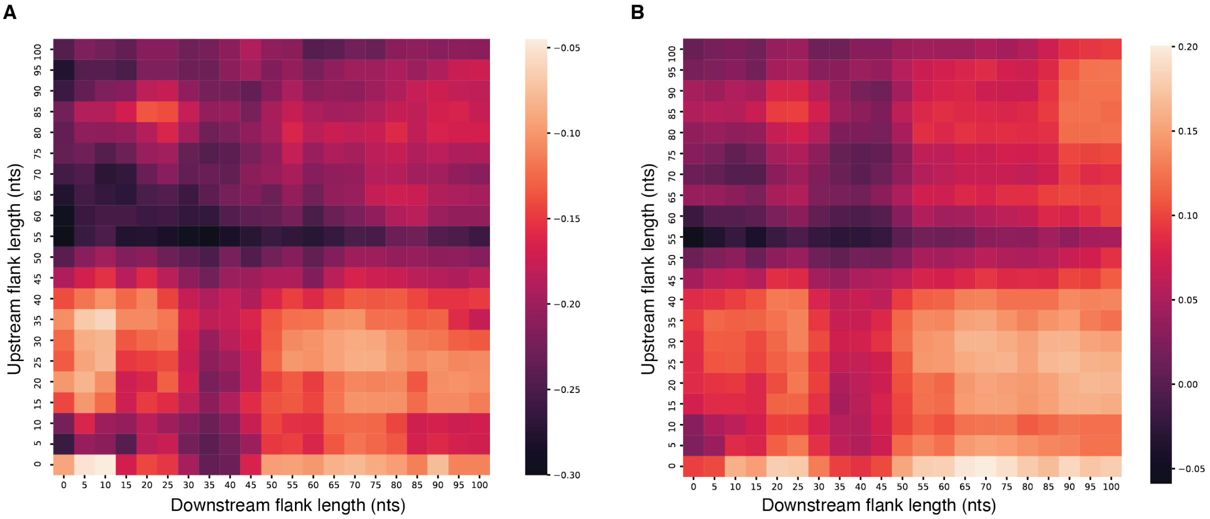

Figure S8. Correlations for various flank lengths around the target site. A, Heatmap showing Spearman correlation between $\Delta G^{\ddagger}_{open}$ and cleavage efficiency for different numbers of flanking nucleotides upstream and downstream of the target site. **B,** Heatmap showing Spearman correlation between $\Delta G^{\ddagger}_{cutting}$ and cleavage efficiency for different numbers of flanking nucleotides upstream and downstream of the target site.

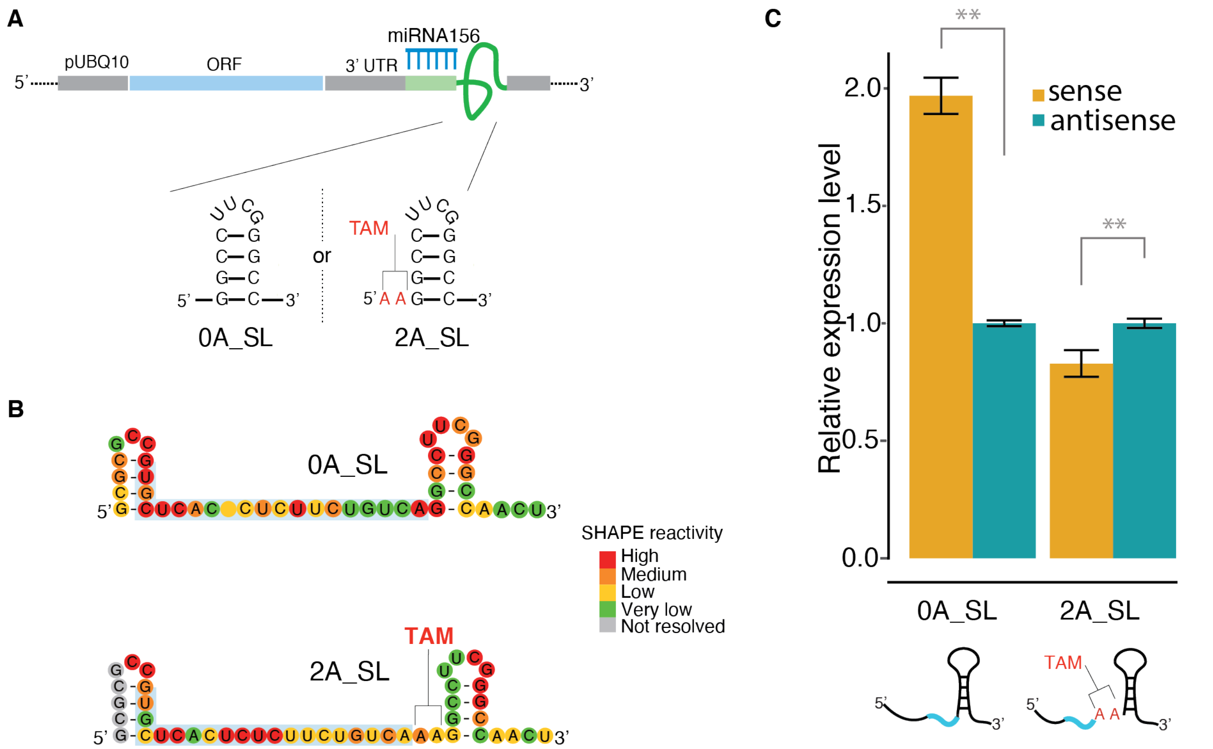

Figure S9. Validation of TAM functionality by a designed structure assay. A, Cartoon representation of the protoplast transformation assay to validate the TAM functionality using a designed structure assay. SL, stem loop structure motif. The miRNA156 target sites (blue comb) followed by 0 or 2 Adenines (As) and ending with a SL. The prefixes, "0A" and "2A", indicate the number of Adenines. **B,** *In vivo* RNA structures of 0A_SL and 2A_SL. **C,** The non-cleaved substrate mRNAs of the structures in **B** was measured by qRT-PCR (dark yellow bars). The antisense target sites were used as controls (teal bars). Data are mean +/- SEM from three independent biological replicates. *P value* < 0.01 by Student's t-test.

Table S1. The list of oligos and primers sequences.

Primer name	Sequence (5'-3')	Experiment
MIR156B-pMDC32-F	taccgggcccccctcgaggcgcgccGCTAGAAGAGGGAGAGATG	Constructs
MIR156B-pMDC32-R	gatcggggaaattcgagctcAGCCAAATTTGAGAGAGAGAG	Constructs
FL-qFP	ATCAGAGAGATCCTCATAAAGGCC	qPCR
FL-qRP	ATTGCCAAATGTTTGAACGATCG	qPCR
RL-qFP	GGAATTATAATGCTTATCTACGTGC	qPCR
RL-qRP	CTTGCGAAAAATGAAGACCTTTTAC	qPCR
miR156-qFP	GCGGCGGTGACAGAAGAGAGT	qPCR
universal-RP	GTGCAGGGTCCGAGGT	Reverse-transcription of miR156/qPCR
miR156-stemloop	GTCGTATCCAGTGCAGGGTCCGAGGTATTCGCACTGGATACGACGTGCTC	Reverse-transcription of miR156
Actin-FP	CAGCCACACTGTCCCAATTTATGAG	binding assay
Actin-RP	TGGATTCCGGCAGCTTCCATTC	binding assay
Firefly_0A_GQS	TCTTTAATTAAATACAAAGGATATCAGGTGGCCCCCGCTGAATTGGAATCGATATTGTTACAACACCCCAACATCTTCGACGCGGGCGTGGCAGGTCTTCCCGACGATGACGCCGGTGAACTTCCCGCCGCCGTTGTTGTTTTGGAGCACGGAAAGACGATGACGGAAAAAGAGATCGTGGATTACGTCGCCAGTCAAGTAACAACCGCGAAAAAGTTGCGCGGAGGAGTTGTGTTTGTGGACGAAGTACCGAAAGGTCTTACCGGAAAACTCGACGCAAGAAAAATCAGAGAGATCCTCATAAAGGCCAAGAAGGGCGGAAAGTCCAAATTGTAGGAGGCGCGCCGTGCTCACTCTCTTCTGTCAGGGAGGGAAGGGGAAGGGGCTGCAGGCTCGAATTTCCCCGATCGTTCAAACATTTGGCAATAAAGTTTCTTAAGATTGAA	Transient expression and binding assay
Firefly_2A_GQS	TCTTTAATTAAATACAAAGGATATCAGGTGGCCCCCGCTGAATTGGAATCGATATTGTTACAACACCCCAACATCTTCGACGCGGGCGTGGCAGGTCTTCCCGACGATGACGCCGGTGAACTTCCCGCCGCCGTTGTTGTTTTGGAGCACGGAAAGACGATGACGGAAAAAGAGATCGTGGATTACGTCGCCAGTCAAGTAACAACCGCGAAAAAGTTGCGCGGAGGAGTTGTGTTTGTGGACGAAGTACCGAAAGGTCTTACCGGAAAACTCGACGCAAGAAAAATCAGAGAGATCCTCATAAAGGCCAAGAAGGGCGGAAAGTCCAAATTGTAGGAGGCGCGCCGTGCTCACTCTCTTCTGTCAAAGGGAGGGAAGGGGAAGGGGCTGCAGGCTCGAATTTCCCCGATCGTTCAAACATTTGGCAATAAAGTTTCTTAAGATTGAA	Transient expression and binding assay
Firefly_0A_SL	TCTTTAATTAAATACAAAGGATATCAGGTGGCCCCCGCTGAATTGGAATCGATATTGTTACAACACCCCAACATCTTCGACGCGGGCGTGGCAGGTCTTCCCGACGATGACGCCGGTGAACTTCCCGCCGCCGTTGTTGTTTTGGAGCACGGAAAGACGATGACGGAAAAAGAGATCGTGGATTACGTCGCCAGTCAAGTAACAACCGCGAAAAAGTTGCGCGGAGGAGTTGTGTTTGTGGACGAAGTACCGAAAGGTCTTACCGGAAAACTCGACGCAAGAAAAATCAGAGAGATCCTCATAAAGGCCAAGAAGGGCGGAAAGTCCAAATTGTAGGAGGCGCGCCGTGCTCACTCTCTTCTGTCAGGCCTTCGGGCCAACTGCAGGCTCGAATTTCCCCGATCGTTCAAACATTTGGCAATAAAGTTTCTTAAGATTGAA	Transient expression and binding assay

Firefly_2A_SL	TCTTTAATTAAATACAAAGGATATCAGGTGGCCCCCGCTGAATTGGAATCGATATTGTTACAACACCCCAACATCTTCGACGCGGGCGTGGCAGGTCTTCCCGACGATGACGCCGGTGAACTTCCCGCCGCCGTTGTTGTTTTGGAGCACGGAAAGACGATGACGGAAAAAGAGATCGTGGATTACGTCGCCAGTCAAGTAACAACCGCGAAAAAGTTGCGCGGAGGAGTTGTGTTTGTGGACGAAGTACCGAAAGGTCTTACCGGAAAACTCGACGCAAGAAAAATCAGAGAGATCCTCATAAAGGCCAAGAAGGGCGGAAAGTCCAAATTGTAGGAGGCGCGCCGTGCTCACTCTCTTCTGTCAAAGGCCTTCGGGCCAACTGCAGGCTCGAATTTCCCCGATCGTTCAAACATTTGGCAATAAAGTTTCTTAAGATTGAA	Transient expression and binding assay
T7_0A_GQS	TAATACGACTCACTATAGGGAGACTCCACCTTCCTACGGTAGTGCTCTCTCTCTTCTGTCAGGGAGGGAAGGGGAAGGGGACCCGACAGTTTTAACTCGTCTGGTTACTCTTAAG	*In vitro* slice assay
T7_2A_GQS	TAATACGACTCACTATAGGGAGACTCCACCTTCCTACGGTAGTGCTCTCTCTCTTCTGTCAAAGGGAGGGAAGGGGAAGGGGACCCGACAGTTTTAACTCGTCTGGTTACTCTTAAG	*In vitro* slice assay
T7_0A_SL	TAATACGACTCACTATAGGGAGACTCCACCTTCCTACGGTAGTGCTCTCTCTCTTCTGTCAGGCCTTCGGGCCAAACCCGACAGTTTTAACTCGTCTGGTTACTCTTAAG	*In vitro* slice assay
T7_2A_SL	TAATACGACTCACTATAGGGAGACTCCACCTTCCTACGGTAGTGCTCTCTCTCTTCTGTCAAAGGCCTTCGGGCCAAACCCGACAGTTTTAACTCGTCTGGTTACTCTTAAG	*In vitro* slice assay
SPL3-T7-FP	TAATACGACTCACTATAGGGACAACTGACTAAAGACGGT	*In vitro* slice assay
SPL3-T7-RP	GAGACACAGAGGATTACAAGGAG	*In vitro* slice assay
T7 -FP	TAATACGACTCACTATAGGGAGACTCC	*In vitro* transcription
T7 -RP	AGTAACCAGACGAGTTAAAACTGTCG	*In vitro* transcription

Table S2. CAP-STRUCTURE-seq constraints improve the structure prediction of structure in the 18S rRNA.

rRNA	Region (nt)	*in silico* vs. phylogenetic structure (PPV/ sensitivity)	Structure-seq[8] vs. phylogenetic structure (PPV/ sensitivity)	CAP-STRUCTURE-seq vs. phylogenetic structure (PPV/ sensitivity)	Idealized constraints vs. phylogenetic structure (PPV/ sensitivity)
18S	Full length	0.27/0.33	0.28/0.33	**0.42/0.41**	0.57/0.58
	98-390	0.34/0.41	0.29/0.34	**0.61/0.54**	0.80/0.75
	392-468	0.38/0.42	0.15/0.16	**0.87/0.68**	0.45/0.53
	608-747	0.74/0.86	0.80/0.86	**0.95/0.86**	0.86/0.86
	1026-1107	0.58/0.63	0.58/0.63	**1.00/0.64**	0.95/1.00
	1480-1579	0.31/0.40	0.30/0.40	**0.58/0.64**	0.79/0.76
	1540-1618	0.33/0.47	0.30/0.47	**0.64/0.82**	0.67/0.82

We used *ViennaRNA* tools version 2.4.9 to predict RNA structure. Our CAP-STRUCTURE-seq is the first SHAPE library in plants, and so contains structural information for all four nucleotide bases. Thus, we compared our new method with our previous DMS Structure-seq data in plants, which contains only A/C structure information (4). For the plant 18S rRNA phylogeny structure, there are several regions that have not been confidently determined due to the lack of co-variation (5). For instance, the region from nucleotide 748 to 869 was left completely single-stranded due to insufficient co-variation evaluation (**Supplementary Figure S6**). This is why the PPV/Sensitivity values of the full length 18S rRNA are not very high, and even with idealized data constraints they only attain 0.57/0.58. However, these values are much higher than the *in silico* prediction. Structure prediction of the 18S rRNA using the CAP-STRUCTURE-seq data yielded improvements not only when compared to the *in silico* prediction but also to the prediction based on DMS Structure-seq data. We further selected six conserved structure regions along the full 18S rRNA(5). Again, the structure predictions using CAP-STRUCTURE-seq data improved on the *in silico* and Structure-seq results for these six regions. Surprisingly, for three of these regions the CAP-STRUCTURE-seq-based predictions improve on the structure predicted by the idealized data. Based on the comparison with the DMS-based Structure-seq data on the 18S rRNA, structure prediction with our CAP-STRUCTURE-seq outperforms DMS Structure-seq. The idealized constraints were produced by mapping paired and single-stranded nucleotides in the phylogenetic structure to SHAPE reactivities of 0 and 1, respectively.

Table S3. The list of CE values for the previously reported target genes.

Target Genes	Gene ID	miRNA	CE (10E-03)
TAS1A	AT2G27400.1	173	497.6
TAS2	AT2G39681.1	173	498.12
TAS1C	AT2G39675.1	173	497.44
TOE2	AT5G60120	172	298.14
LAC4	AT2G38080.1	397	207.66
GUN5	AT5G13630.1	395	198.59
LCR	AT1G27340.1	394	188.75
AFB2	AT3G26810.1	393	121.48
AFB3	AT1G12820.1	393	111.48
AFB1	AT4G03190.1	393	107.04
APS4	AT5G43780.1	395	67.77
ARF10	AT2G28350.1	160	40.95
ARF16	AT4G30080.1	160	40.94
TIR1	AT3G62980.1	393	38.51
NF-YA3	AT1G72830.2	169	38.19
LOM1	AT2G45160.1	170	25.6
		171	17.72
SCL6-IV	AT4G00150.1	170	25.04
		171	17.34
LOM2	AT3G60630.1	170	24.64
		171	17.06
CIB4	AT1G10120.1	396	2.82
SPL2	AT5G43270	156	2.4
APS1	AT3G22890.1	395	1.02
PHV	AT1G30490.1	166	0.94
		165	0.52
REV	AT5G60690.1	166	0.9
		165	0.49
MYB65	AT3G11440.1	159	0.75
		319	0.48
MYB33	AT5G06100.2	159	0.73
		319	0.47
ATHB-8	AT4G32880.1	166	2.45E-07
		165	1.35E-07
ARF8	AT5G37020.1	167	0
NF-YA9	AT3G20910.1	169	0
AP2	AT4G36920	172	0
SNZ	AT2G39250	172	0
TCP24	AT1G30210.1	319	0
TCP2	AT4G18390.1	319	0

AST68	AT5G10180.1	395	0
APS3	AT4G14680.1	395	0
GRF9	AT2G45480.1	396	0
Metallopeptidase	AT3G05350.1	397	0
SUMM2	AT1G12280.1	472	0
CC-NBS-LRR family	AT5G43740.1	472	0

Supplementary Methods. The detailed calculation of miRNA cleavage efficiency.

Combining miRNA-seq, RNA-seq and degradome libraries to estimate *in vivo* miRNA cleavage efficiency

RNA degradation is relatively efficient (6) and there are many different pathways that can generate degradation products in the degradome library besides miRNA cleavage, for example, deadenylation-mediated mRNA decay, non-sense mediated decay (NMD) or XRN4-mediated co-translational mRNA decay (reviewed by (7)). miRNAs regulate mRNAs through translational repression, mRNA de-stabilization and mRNA cleavage. In animals, it has been suggested that translational repression is prevalent, which can then be followed by de-stabilization, such as shortening of the poly-A tails at the 3' end and removal of the cap at the 5' end (8). In plants, cleavage is the dominant pathway for miRNAs to regulate their target mRNAs (9). In order to compare the ability of different miRNAs to cleave their targets on a global scale, we need to quantify the cleavage efficiency (CE) of miRNAs at their target sites.

Our CE calculation is based on two underlying facts (2, 10): miRNA-mediated cleavage is the major mRNA turnover pathway for target genes; the 5' cleaved products are located within binding sites, which are temporally stable. Therefore, the degradation signal within target sites reflects the cleavage products from miRISC cleavage. These two facts were also confirmed by our analysis below.

Firstly, to confirm that the degradation signal within target sites is mainly from miRNA-mediated cleavage, we mapped the 5'end of our WT *A. thaliana* degradome reads to previously validated cleavage sites (2). We found that most of the read ends were mapped at the tenth nucleotide of the miRNA complementary sites (Supplementary Figure S3C), which provides strong evidence of miRNA cleavage, as other degradation pathways rarely prefer to leave the 5' cleavage end exactly at the tenth position of miRNA complementary sites. Additionally, to confirm that the 5' cleaved products are temporally stable, we mapped the read ends of the cleavage products in the *xrn4* mutant to previously validated cleavage sites (2). The cleavage site distribution in *xrn4* mutant exhibited the same pattern as WT (Supplementary Figure S3D), which is consistent with the notion that miRNA cleavage products are temporally stable intermediates, resistant to cellular XRN4 exonuclease in *A. thaliana*, although the precise mechanism is currently unknown (10, 11). Thus, we counted the degradation reads within target sites as the outcomes of cleavage products from miRISC cleavage.

Since AGO1 and miRNA are an enzyme complex, we defined the cleavage efficiency (CE) in a similar way to enzyme activity. In detail, the catalytic ability of an enzyme can be defined as the amount of product generated by one unit of enzyme from one unit of substrate, which led us to define:

$$\boldsymbol{CE = \frac{Number\ of\ miRNA\ mediated\ cleavage\ products}{Total\ number\ of\ miRNA\ target\ transcripts \times\ Number\ of\ miRNAs}}.$$

Cleaved transcripts can be characterized by the 5' phosphate featured on 3' cleavage products. We constructed a degradome library to capture cleavage products. RNA abundance of degradation products can be measured by the Reads Per Kilobase of transcript per Million mapped reads (RPKM). RPKM is a measure of relative RNA concentration in the whole transcriptome. In the degradome, the RPKM of each mRNA means relative degraded mRNA fragment number compared to all degradation products, i.e.,

$$\boldsymbol{Degradome[RPKM] \approx \frac{Number\ of\ cleavage\ products}{Total\ degradation\ products}}.$$

To quantify the miRNA-mediated degradation products, we counted and designated the reads mapped within each target site as the products of miRNA-mediated cleavage. Therefore, we can label the miRNA-mediated Degradome RPKM as mirDegradome[RPKM], whereby:

$$\boldsymbol{mirDegradome[RPKM] \approx \frac{Number\ of\ miRNA\ mediated\ cleavage\ products}{Total\ degradation\ products}}.$$

Similar to the degradome, the RPKM of each mRNA can be described as,

$$\boldsymbol{RNAseq[RPKM] \approx \frac{Total\ number\ of\ miRNA\ target\ transcripts}{Total\ RNA\ fragments}}.$$

The miRNA RPKM estimate can be derived from the miRNA library. Similarly,

$$\boldsymbol{miRNAseq[RPKM] \approx \frac{Number\ of\ miRNAs}{Total\ miRNA\ products}}$$

so

$$\boldsymbol{CE \approx \alpha * \frac{mirDegradome[RPKM]}{RNAseq[RPKM] \times miRNAseq[RPKM]}}$$

where

$$\boldsymbol{\alpha = \frac{Total\ degradation\ products}{Total\ RNA\ fragments \times Total\ miRNA\ products}}$$

The total degradation products, total RNAs and total miRNAs should be constant and be reflected by the library sequencing depth. Therefore, α is a constant.

The population of each mRNA is constant over time due to the dynamic equilibrium of an intact mRNA and its degraded products (12). Therefore,

$$\boldsymbol{Total\ number\ of\ mRNA} \\ = \boldsymbol{Number\ of\ intact\ mRNA} + \boldsymbol{Number\ of\ degraded\ mRNA}$$

In our study, (-)SHAPE library can estimate the intact mRNA abundance. Indeed, we found that RNAseq[RPKM] was tightly correlated with (-)SHAPE[RPKM] +Degradome[RPKM] (Supplementary Figure S5E), i.e.,

$$\boldsymbol{RNAseq}[\boldsymbol{RPKM}] \approx \beta((-)\boldsymbol{SHAPE}\ [\boldsymbol{RPKM}] + \boldsymbol{Degradome}[\boldsymbol{RPKM}]),$$

in which β is a constant. The advantage of combining the (-)SHAPE and the degradome libraries to calculate the CE lies in its focus on miRNA-mediated cleavage events. Then, for miRNA target genes,

$$\boldsymbol{RNAseq}[\boldsymbol{RPKM}] \approx \beta((-)\boldsymbol{SHAPE}\ [\boldsymbol{RPKM}] + \mathbf{m}\boldsymbol{irDegradome}[\boldsymbol{RPKM}]),$$

thus

$$\boldsymbol{CE} \approx \frac{\boldsymbol{\alpha}}{\boldsymbol{\beta}} * \frac{\boldsymbol{mirDegradome}[\boldsymbol{RPKM}]}{((-)\boldsymbol{SHAPE}\ [\boldsymbol{RPKM}] + \boldsymbol{mirDegradome}[\boldsymbol{RPKM}]) \times \boldsymbol{miRNAseq}[\boldsymbol{RPKM}]}$$

or

$$CE \propto \frac{\boldsymbol{mirDegradome}[\boldsymbol{RPKM}]}{\big((-)\boldsymbol{SHAPE}\ [\boldsymbol{RPKM}] + \boldsymbol{mirDegradome}[\boldsymbol{RPKM}]\big) \times \boldsymbol{miRNAseq}[\boldsymbol{RPKM}]}.$$

REFERENCES

1. Fahlgren,N., Howell,M.D., Kasschau,K.D., Chapman,E.J., Sullivan,C.M., Cumbie,J.S., Givan,S.A., Law,T.F., Grant,S.R., Dangl,J.L., *et al.* (2007) High-throughput sequencing of Arabidopsis microRNAs: Evidence for frequent birth and death of MIRNA genes. *PLoS One*, **2**.
2. Addo-Quaye,C., Eshoo,T.W., Bartel,D.P. and Axtell,M.J. (2008) Endogenous siRNA and miRNA Targets Identified by Sequencing of the Arabidopsis Degradome. *Curr. Biol.*, **18**, 758–762.
3. Weeks,K.M. and Crothers,D.M. (1991) RNA recognition by Tat-derived peptides: Interaction in the major groove? *Cell*, **66**, 577–588.
4. Ding,Y., Tang,Y., Kwok,C.K., Zhang,Y., Bevilacqua,P.C. and Assmann,S.M. (2014) In vivo genome-wide profiling of RNA secondary structure reveals novel regulatory features. *Nature*, **505**, 696–700.
5. Cannone,J.J., Subramanian,S., Schnare,M.N., Collett,J.R., D'Souza,L.M., Du,Y., Feng,B., Lin,N., Madabusi,L. V, Müller,K.M., *et al.* (2002) The comparative RNA web (CRW) site: an online database of comparative sequence and structure information for ribosomal, intron, and other RNAs. *BMC Bioinformatics*, **3**, 2.
6. Houseley,J. and Tollervey,D. (2009) The Many Pathways of RNA Degradation. *Cell*, **136**,

763–776.
7. Zhang,X. and Guo,H. (2017) mRNA decay in plants: both quantity and quality matter. *Curr. Opin. Plant Biol.*, **35**, 138–144.
8. Djuranovic,S., Nahvi,A. and Green,R. (2012) miRNA-mediated gene silencing by translational repression followed by mRNA deadenylation and decay. *Science*, **336**, 237–40.
9. Chen,X. (2009) Small RNAs and Their Roles in Plant Development. *Annu. Rev. Cell Dev. Biol.*, **25**, 21–44.
10. Jackowiak,P., Nowacka,M., Strozycki,P.M. and Figlerowicz,M. (2011) RNA degradome-its biogenesis and functions. *Nucleic Acids Res.*, **39**, 7361–7370.
11. Llave,C., Xie,Z., Kasschau,K.D. and Carrington,J.C. (2002) Cleavage of Scarecrow-like mRNA Targets Directed by a Class of Arabidopsis miRNA. *Science (80-.).*, **297**, 2053–2056.
12. Miller,C., Schwalb,B., Maier,K., Schulz,D., Dümcke,S., Zacher,B., Mayer,A., Sydow,J., Marcinowski,L., Dölken,L., *et al.* (2011) Dynamic transcriptome analysis measures rates of mRNA synthesis and decay in yeast. *Mol. Syst. Biol.*, **7**, 458.
